# *QuickStats:* Age-Adjusted Death Rates* for Alzheimer Disease^†^ Among Adults Aged ≥65 Years, by Sex — National Vital Statistics System, United States, 1999–2019

**DOI:** 10.15585/mmwr.mm7016a5

**Published:** 2021-04-23

**Figure:**
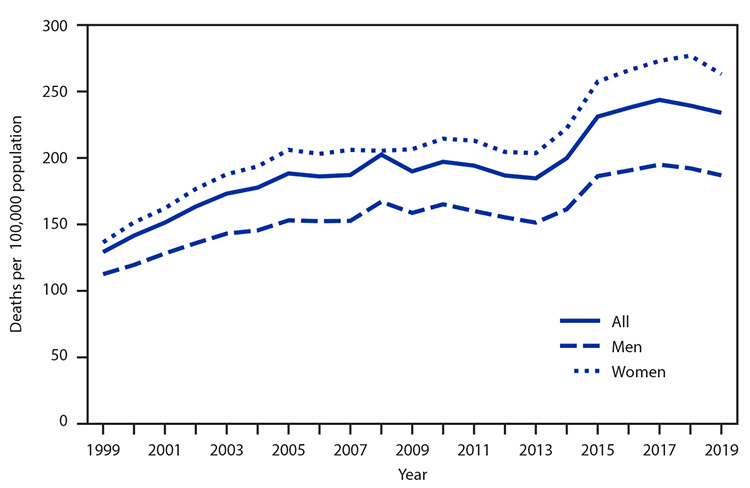
The age-adjusted death rate for Alzheimer disease increased from 128.8 per 100,000 in 1999 to 233.8 in 2019. The trend for the total population and for men and women alternated between periods of general increase and periods of stability. Rates were stable from 2016 to 2019, and in 2019 were 263.0 for women and 186.3 for men. Throughout the 1999–2019 period, the rate was higher for women than for men.

**Source:** National Center for Health Statistics, National Vital Statistics System, Mortality Data, 1999–2019. https://www.cdc.gov/nchs/nvss/deaths.htm

